# Effect of antibacterial/adhesive approaches on bonding durability of fiber posts cemented with self-etch resin cement

**DOI:** 10.4317/jced.53992

**Published:** 2017-09-01

**Authors:** Fereshteh Shafiei, Mahtab Memarpour, Narges Vafamand, Mahsa Mohammadi

**Affiliations:** 1 DMD, MScD, Professor, Oral and Dental Disease Research Center, School of Dentistry, Shiraz University of Medical Sciences, Shiraz, Iran; 2 DDS, Dentist, Department of Operative Dentistry, School of Dentistry, Shiraz University of Medical Sciences, Shiraz, Iran

## Abstract

**Background:**

Longevity of post-retained restoration is highly depended on bonding stability of fiber post (FP) to root dentin. This study evaluated the effect of different antibacterial/adhesive approaches on bonding durability of FPs luted into root canal with a self-etch cement.

**Material and Methods:**

Seventy-two human maxillary central incisor roots were divided into six groups after endodontic treatment, based on the antibacterial/adhesive treatments as follows: 1)ED primer II (ED, control); 2) Clearfil Protect Bond (PB); 3) 2% chlorhexidine (CH) pretreatment + ED primer II (CH+ED); 4) CH-incorporated into ED primer II (CH in ED); 5) CH pretreatment + Clearfil SE Bond (CH+SE); and 6)CH-incorporated into SE primer (CH in SE). The FPs were then cemented using PanaviaF2.0. After micro-slicing the bonded root dentin, a push-out bond strength (PBS) test was performed immediately or after two years of water storage. Data were analyzed using ANOVA and post hoc Tukey tests (α=0.05).

**Results:**

The effects of antibacterial/adhesive approach, time and interaction between the main factors were significant (*p*=0.01). There was no significant difference between the immediate groups, except between the CH+ED group (the lowest PBS) and PB and CH in SE groups (the highest PBS) (*p*≤0.03). After aging, the same difference was observed (*p*≤0.02); the control group exhibited a significantly lower PBS compared to the other groups (*p*≤0.01), except for CH+ED. Aging significantly decreased PBS of all the groups (*p*≤0.01); the control group exhibited the highest reduction.

**Conclusions:**

CH incorporated into self-etch primers or in pretreatment step prior to two-step self-etch adhesive and antibacterial adhesive could improve bond stability of self-etch cemented fiber post. However, none of these was capable of inhibiting bond degradation over time.

** Key words:**Push-out bond strength, Fiber post, Chlorhexidine.

## Introduction

Numerous favorable properties of fiber posts (FP) have resulted in their widespread use for the restoration of endodontically treated teeth. In adhesive cementation of FPs establishment of a highly durable bond between resin cement and root dentin is an essential factor to provide a coronal seal and adequate retention (1).

Self-etch adhesive (SE) resin cements might be preferred to etch-and-rinse ones by clinicians due to simplification and less technique sensitivity (2). These systems do not need acid etching and rinsing. As a result, a thick and heavy secondary smear layer formed during post space preparation could remain, which possibly contains microorganisms. These, in addition to penetration of oral bacteria through coronal leakage, might jeopardize prognosis of root canal therapy (3). Therefore, use of antibacterial agents is a critical and important step during post luting procedures. Chlorhexidine (CH) with excellent substantive antibacterial properties acts as a matrix-metalloproteinase (MMP) and cathepsin inhibitor, preserving collagen matrix of dentin (4,5). Some studies reported bonding longevity of adhesive-cemented FPs following CH irrigation of post space in root canals with different results (6-8). However, this approach introduces an additional step to complex fiber post adhesive cementation, increasing chair-time. Although this paves the way for benefiting from better efficacy of antibacterial activity of CH in 2% concentration than lower concentrations (9) in the root canal, it might limit the efficacy of MMP inhibitory activity of CH due to simultaneous demineralization and resin penetration of SE cements into the smear layer-covered dentin.

On this basis, incorporating CH into acidic primers could be another approach to provision of positive effects of CH during adhesive cementation. Firstly, Hiraishi et al. demonstrated that incorporation of 1% CH into ED Primer 2.0 exhibited significant antibacterial activity without any adverse effect on bond strength of Panavia F2.0 to dentin (10). This procedure could dissolve/infiltrate the smear layer along with CH penetration, consequently allowing facilitated interaction of CH with the activated MMPs by acidic monomers and inhibitory effect of CH. This approach was found to have positive results in bonding durability of FPs bonded with Panavia F2.0 (11).

Quaternary ammonium compounds (QAC), as a main antibacterial agent incorporated into the adhesive systems, have a stable and durable antibacterial activity (12). MDPB is the combined QAC, and methacrylate group as an antibacterial monomer has a disinfecting ability in an uncured state. After copolymerization with other monomers, it inhibits bacterial growth on its surface (12,13). The more antibacterial effectiveness of experimental solution containing MDPB than CH solution on bacteria related to endodontic infections has been clarified by a recent study (14). This monomer has been incorporated into a commercially available two-step SE, Clearfil Protect Bond by Kuraray (12). This adhesive exhibited a stable bond strength to coronal dentin after one year and better antibacterial activity compared to the other antibacterial agents incorporated (CH and glutaraldehyde) (15). Recently MDPB has been found to inhibit MMPs and cathepsins (16).

To date, no study has compared bonding longevity of SE cement to intraradicular dentin following different antibacterial applications during fiber post cementation.

The aim of this study was to test the null hypothesis that various adhesive/antibacterial approaches have no effects on bonding performance of an SE cement in root canal space immediately and at long term.

## Material and Methods

Seventy-two sound human maxillary central incisors with approximately similar size and anatomic shape were selected. The roots of the selected teeth were free of cracks and root resorption and were mature. They were stored in 0.5% chloramine-T solution at 4°C and then in distilled water. They were used following informed consent from the patients and approval of the study protocol by the local Ethics Committee. The roots were cut to obtain a uniform length of 15 mm from the apex and then endodontically treated.

After one week of storage in water, post spaces were prepared to create a standardized depth of 10 mm using the respective drills provided by the post manufacturer. These procedures were verified by radiographs.

FPs (Glassix Post, H.Nordin, SA, Chailly-Montreux, Switzerland) were tried in the prepared canals for a passive fit. The post surfaces were cleaned with ethanol, air-dried and then silanized. The specimens were then randomly divided into six groups (n=12) based on the antibacterial/adhesive treatments of the root dentin as follows.

Group 1 (ED): ED primer II was applied as a control.

Group 2 (PB): A two-step SE antibacterial adhesive, Clearfil Protect Bond, was applied.

Group 3 (CH+ED): 2% chlorhexidine (CH) solution (Consepsis, Ultradent, USA) was applied using an endodontic brush for 60 s. Then the canals were dried with paper points and ED primer II was applied.

Group 4 (CH in ED): Chlorhexidine diacetate (Sigma-Aldrich, St. Louis, MO ,USA) was directly added to a mixture of ED primer II A and B to prepare the primer containing 1 wt%.

Group 5 (CH+SE): CH solution was applied similar to that in group 3. Then, a two-step SE (Clearfil SE Bond) was applied.

Group 6 (CH in SE): The CH-incorporated Clearfil SE primer was prepared similar to that in ED primer and then Clearfil SE Bond was applied.

During adhesive post cementation, the roots were held in a moist gauze sponge to maintain their moisture content. All the bonding steps were carried out by the same operator, according to manufacturer’s instructions ( Table 1). In all the groups, the mixed Panavia F2.0 was applied on the post surface and to the post space. The post was immediately seated with a slight vibratory motion and held under finger pressure; after removal of the excess cement, light-curing was performed for 60 s at 600 mW/cm2 using a light-curing unit (VIP Junior, Bisco, Schaumburg, IL, USA). After one week of water storage, each root was sectioned to obtain seven 1-mm-thick slices by using a slow-speed cutting machine (Mecatome T201 A, Presi, Grenoble, France). The first coronal slice was excluded. In half of the roots from each group (n=6, 36 slices), the push-out test was performed immediately. The other half of each group was stored in distilled water containing 0.4% sodium azide for two years before assessing the long-term bond strength.

**Table 1:**
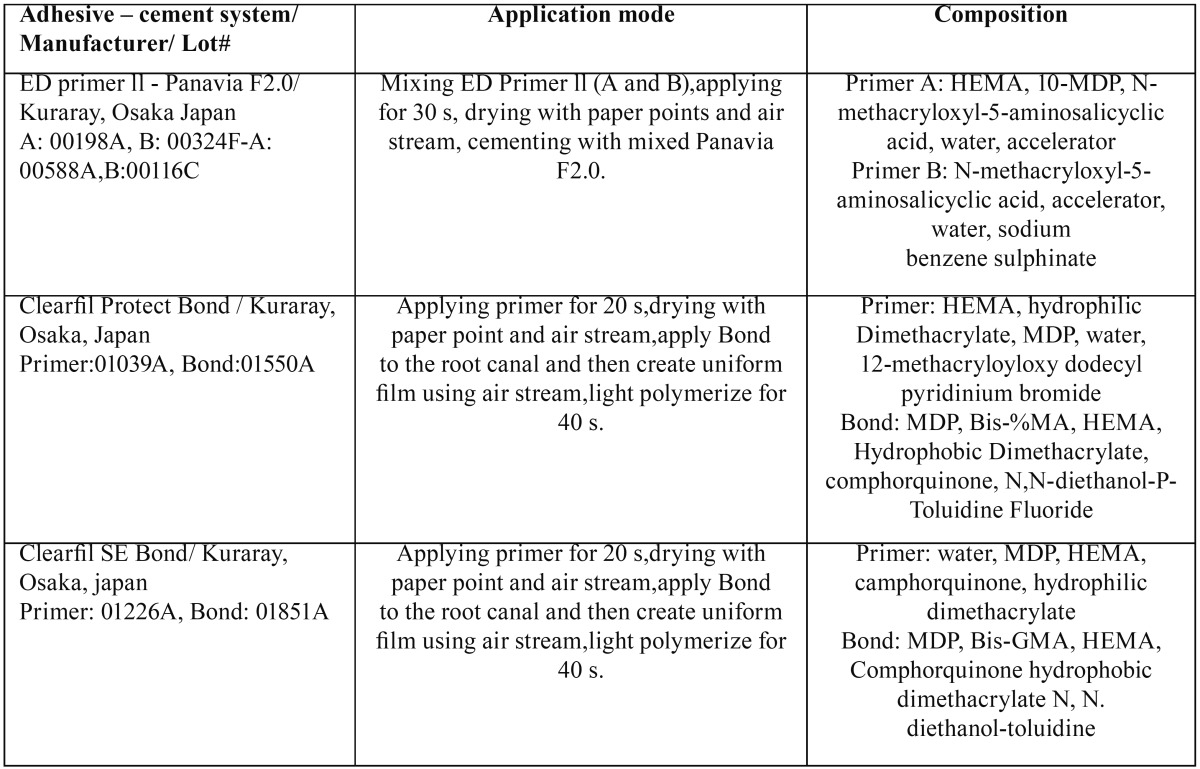
Materials and application procedures used in the current study.

The slices were submitted to a compressive load in a universal testing machine (Zwick, Roell, Ulm, Germany) at 0.5 mm/min on the center or the apical surface of the post in an apico-coronal direction until the shear stress applied along the bonded interface dislodged the post. The load of debonding in Newton (N) was divided by the adhesive interface area and push-out bond strength was recorded in MPa. The bonded area was calculated through the following formula π(R+r)[(h2+(R-r)2],0.5 where R and r represent the coronal and the apical root canal radii, respectively, and h is the thickness of the slice.

All the debonded root slices were assessed under a stereomicroscope (Carl Zeiss Inc, Oberkochen, Germany) at ×40 and classified as follows: 1) adhesive failure in the dentin; 2) cohesive failure in the cement; 3) adhesive failure between the dentin and cement; 4) adhesive failure between the post and cement; and 5) mixed failures consisting of a combination of two or more failure modes.

**Figure 1:**
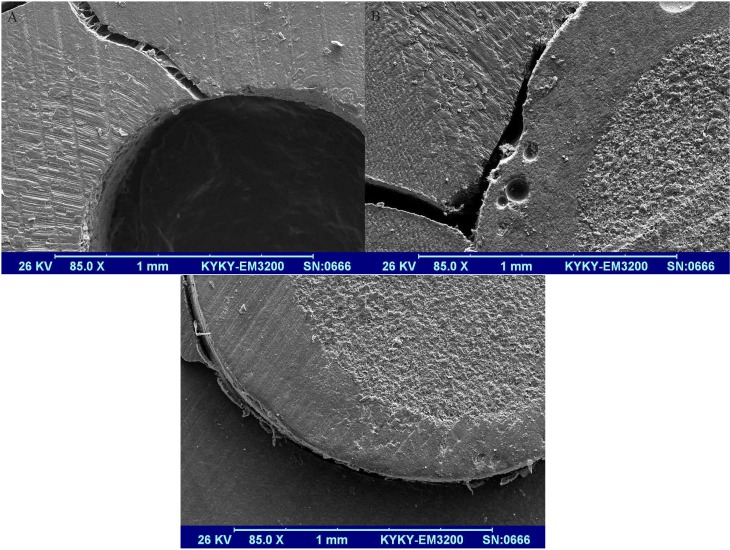
Scanning electron micrograph of representative failure modes: A and B) Mixed failures including adhesive failure between the resin cement and root dentin along with cohesive failure in the root dentin, (A): showing no remaining resin on root dentin wall and (B): showing a gap-free interface between the resin cement and post. C) Adhesive failure between the resin cement and the root dentin.

The representative specimens of failure modes were prepared for scanning electron microscopy (SEM; EM3200, KYKY, Beijing, China) observations of the failure patterns as shown in Figure 1.

## Results

 Table 2 presents the means and standard deviations (in MPa) of push-out bond strengths (PBS) of the six groups. Two-way ANOVA showed that the effects of adhesive/antibacterial agent, time and interaction between the two main factors were significant (*p*≤0.01).

**Table 2:**
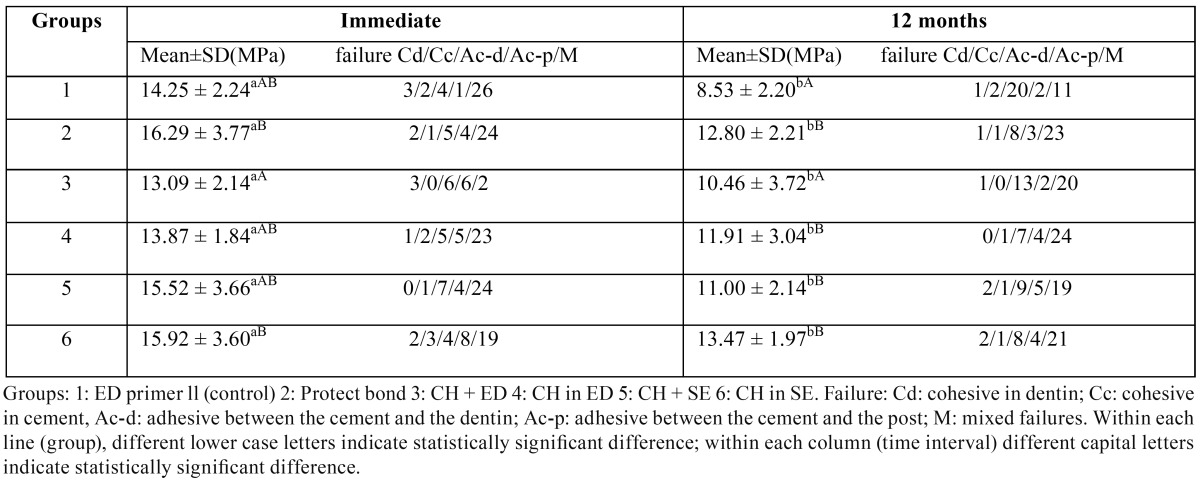
Push-out bond strength (mean ± SD) of Panavia F2.0 in the six groups at two time intervals and failure mode (n=36).

For each time interval, one-way ANOVA and post hoc Tukey tests were used to compare the PBS of the six groups. Student’s t-test was used to compare the effect of aging on PBS in each group (α=0.05).

CH+ED (13±2.1) exhibited a lower immediate PBS compared to those of PB (16.29±3.7) and CH in the SE (15.92±3.6) groups (*p*=0.01 and *p*=0.03, respectively). There was no significant difference between the other groups (*p*>0.05).

After aging, PBS of the ED control group (8.53±2.2) was significantly lower than those of the other groups (*p*≤0.01), except for the CH+ED group (10.46±3.7). Similar to the immediate PBS group, the latter group yielded a lower PBS than those of the PB group (12.8±2.2) and CH in the SE group with the highest PBS (13.47±1.9) (*p*=0.02 and *p*=0.002, respectively). PBS of the latter group was significantly higher than the PBS of CH+SE group (11±2.1) (*p*=0.01).

Aging significantly decreased PBS of all the groups with different extents (*p*≤0.01). The highest absolute reduction was recorded for the control (ED) group (11.31), followed by the CH+SE (4.95) and PB (3.64) groups. The other three groups exhibited a comparable absolute reduction (2.8).

The results of failure analysis of the six study groups revealed that the majority of failure modes were mixed failures in immediate groups. After aging the modes of failure were mostly adhesive failures between the root dentin and cement in the ED primer (control) group, while in the other groups mixed failure was the predominant failure mode ( Table 2).

## Discussion

According to the results of this study, there was a significant difference between immediate PBS of different adhesive/antibacterial procedures, rejecting part of the tested null hypothesis. Although incorporation of CH into ED primer/SE primer (1%) or its separate application (2%) had no significant effect on initial bonding ability of Panavia F2.0, separate application of CH before ED primer led to significantly lower PBS compared to those of only PB and CH in the SE groups. A similar significant difference was observed after aging. These two groups and CH+SE with immediate comparable PBS (16.29‒15.52) are two-step SE systems that have a separately applied resin layer with higher hydrophobicity. It was reported that the additional light-cured resin layer applied on ED primer might provide additional free radicals to improve the rate and extent of polymerization of ED primer and contribute to reduced permeability of the adhesive layer (17). ED primer is a single-step SE adhesive that exhibits some permeability due to its high content of hydrophilic and acidic monomer components and the lack of the subsequent application of a hydrophobic resin layer (17). In vivo permeability of these simplified adhesives was previously documented even in root-treated dentin. This might negatively influence bonding of dual-cured resin cement to the root dentin. This permeability could also occur in vitro even without a perfusion system because the teeth were retrieved from the water storage medium and bonded in their normal hydrated state (18). It was suggested that ED primer is essential for adequate polymerization of Panavia F; even in the absence of light-curing, not for bonding to dentin.

Another explanation for improved bond strength of Panavia F2.0 to coronal dentin after adding the resin layer reported by Carvarlho *et al.* was the relief of shrinkage stress induced by the resin cement (17). The positive effect of this factor could be more relevant in deep, narrow and confined root canal space with the extremely high C-factor.

Our results revealed that direct incorporation of 1% CH into ED primer had no adverse effect on immediate PBS. A similar result was reported by Zhou *et al.* during bonding of FP with Panavia F2.0 to root dentin (11). Zhou *et al.* reported no adverse effects of adding CH at≤1 wt% into self-etch primer of Clearfil SE Bond on the immediate bond strength. These authors demonstrated that this combination could preserve the bond strength to coronal dentin after 12 months of water aging (19). This positive effect was related to the MMP inhibitory effect of incorporated of CH into SE primer (20). No study has evaluated this approach in root canals.

Based on the results of the current study, after aging the coronal group (ED primer) exhibited a significantly lower PBS compared to those of the other groups with antibacterial agent except for the CH+ED group. In this context, the diminished PBS observed in all the groups was the highest for ED primer group. Therefore, the other part of the tested hypothesis could not be confirmed.

Two main factors are involved in adhesive bond degradation: collagen degradation and hydrolysis of hydrophilic ionic resin monomers in the simplified primer/adhesive (21,22). The results of this study might be attributed to the factors mentioned above during the accelerated aging via direct water exposure of micro-sliced specimens. A rapid degradation process might have occurred following rapid water diffusion through the small surface area of the adhesive interface (22). In particular, difficulties in bonding and penetration of curing light into root canal compared with coronal dentin could create a weak polymer with low degree of polymerization (11), enhancing resin degradation. The highest PBS was obtained in CH in the SE group, which was significantly higher than that in the CH+SE group. This might be attributed to the partial protective effect of CH on bonding longevity through an inhibitory effect on MMPs. This effect appeared to depend on application mode of CH with each adhesive so that CH incorporated into Clearfil SE primer was more effective than separately applying CH before SE primer. The curing of a hydrophobic resin layer on the hybrid layer treated with CH can cover and preserve CH at the adhesive interface to prolong its beneficial effects (23).

Hiraishi *et al.* demonstrated that CH pretreatment might be an adverse effect on immediate bonding performance of Panavia F2.0 cement (24). These authors believed that this might be attributed to the moisture control in the coronal dentin treated with CH rather than to the intrinsic properties of CH (24). The incorporation of CH into primer can simplify bonding procedures along with lower technique sensitivity. This aspect might have partly contributed to our results in relation to the ineffectiveness of CH application prior to ED primer in diminishing PBS loss over time. Lindblad *et al.* found no effect of CH pretreatment on immediate and one-year PBS of FPs with ER and self-adhesive resin cements in root canal space, but CH changed the fracture pattern from pure adhesive-to-dentin failure to mixed and cohesive-in-dentin failures (25,26). In the current study, despite similar failure modes in the immediate groups, distribution of failure modes was different among the groups after aging. In this context, in the control group the adhesive failure between the cement and root dentin was predominant, while mixed failure was dominant for the other groups, possibly indicating the positive effect of CH and the antibacterial effect of adhesive on bonding longevity. Conflicting results have been reported regarding MMP-inhibitory activity of MDPB and bond stability of the antibacterial adhesive (27). Consistent with our results, some studies have shown no adverse effects of 2% CH pretreatment on immediate PBS of posts bonded with SE adhesive cements (6,28). However, an adverse effect of CH on CSE, especially in the apical third of the root, was reported (2). The beneficial effect of CH on preserving PBS of fiber post bonded with SE adhesive (CSE) was confirmed in two one-year studies (7,8). Contrary to our results, in these studies PBS of CH-pretreated groups did not significantly decrease after one year; the full length of bonded roots were water-aged and then submitted to slice preparation for PBS testing. However, the bonded roots of the current study were first sectioned and then water-aged. The different aging processes used might explain the differences in the results.

Although long-term water storage of root micro-slices and the subsequent push-out test cannot closely mimic clinical aging, this experimental set-up was previously designed to evaluate the role of anti-MMP property of CH and the other agent on bonding stability of FPs to radicular dentin (11,29). Furthermore, similar patterns of hybrid layer degradation have been found in vivo from the base of adhesive-bonded cavities (30). A study by Zhou *et al.* on CH showed the positive effect of 1% CH incorporated into ED primer on bonding durability of FPs for 18 months of water storage. However, a significant decrease in PBS was observed in the 1% CH group (11). This finding was supported by the results of the current study.

The long-term effect of CH might be attributed to its high substantivity and its electrostatic binding to the mineralized and demineralized dentin (30). Nevertheless, CH might be released from the adhesive interface over time, decreasing its anti-MMP effect (20). This may be higher in this experimental set-up than in vivo condition due to a higher direct contact of sectioned adhesive interface with water during water storage. The antibacterial adhesive does not release the antibacterial component over time. This study was conducted using single SE resin cement. The adhesives used instead of ED primer II were from the same manufacturer as Panavia F2.0, preventing any incompatibility between different products. The results of the present study cannot be generalized to other adhesive resin cements with different chemistry.

## Conclusions

Considering the limitations of this in vitro study, it can be concluded that CH incorporated into self-etch primers or in pretreatment step prior to application of Clearfil SE Bond and antibacterial adhesive could improve bond stability of FPs in root canal space. Nevertheless, none of these antibacterial protocols was capable of completely inhibiting bond degradation in the long term.
